# Comparison of aloe vera gel dressing with conventional dressing on pressure ulcer pain reduction: a clinical trial

**DOI:** 10.1186/s13104-023-06682-8

**Published:** 2024-01-16

**Authors:** Azam Malek Hosseini, Mohammad Rostam Khani, Sina Abdi, Siavash Abdi, Nader Sharifi

**Affiliations:** 1 Department of Nursing, Khomein University of Medical Sciences, Khomein, Iran; 2 Department of Medicine, Faculty of Medicine, Arak University of Medical Sciences, Arak, Iran; 3 Department of Public Health, Khomein University of Medical Sciences, Khomein, Iran

**Keywords:** Pressure Ulcer, Pain, Aloe vera Gel

## Abstract

**Background:**

Aloe Vera has a strong analgesic and anti-inflammatory effect, and its use effectively controls pain. This study aimed to determine the effect of Aloe Vera gel versus saline on pain relief of pressure ulcers.

**Methods:**

This study is a double-blind, randomized clinical trial conducted in Valiasr Hospital in Arak, Markazi Province, in the center of Iran from May 2020 to April 2022. Using the available sampling method, 95 patients with pressure ulcers were assessed for eligibility, 64 patients were selected, 33 patients were placed in the experimental and 34 patients in control. For the experimental group, the ulcers were first cleaned with normal saline to remove the slough and then the already prepared Aloe Vera gel was evenly applied. For control groups, the ulcers were first washed with normal saline to remove off the slough and then covered with a sterile cotton gage, and the wound was tightly bandaged with a cotton roll to keep the “daily dressing” in place. A visual pain scale was used to assess the patient’s pain level. Data analysis was done using SPSS 17. Descriptive statistics, ANOVA, and greenhouse tests were used. The significance level was 0.05.

**Results:**

The results showed that the average pain score in both groups had a downward trend; that is, both dressings effectively reduced pressure ulcer pain (*P* < 0.001). The greenhouse test results showed that the difference between the two groups was significant (*P* < 0.001).

**Conclusion:**

The results showed the effect of Aloe Vera gel in reducing pressure ulcer pain. Dressing with Aloe Vera gel is preferable to reducing pain during dressing changes in patients with pressure ulcers.

**Trial registration:**

Iranian Registry of Clinical Trials IRCT20180715040478N2, 2021-08-17.

## Introduction

Pressure or skin ulcers are chronic and prevalent in older adults and healthcare settings. Pressure ulcer is one of the common characteristics of people with little or no mobility, which is caused by the pressure of body weight on skin areas for long periods [1]. Worldwide, more than 60,000 patients die from severe pressure ulcers every year [2].

Pressure ulcers range in severity from skin erythema without blanching (Category I), superficial skin loss (Category II), and large ulcers involving fat, muscle, and bone (Category III/IV) [3]. These ulcers occur at home and are widely prevalent in all healthcare settings. They are also associated with more extended hospital stays and thus higher healthcare costs and have a major impact on health-related quality of life (HRQL) [4–7]. The pain and discomfort caused by these wounds are a common problem for patients and the healthcare system [8–10]. Chronic pain can have a major adverse effect on the lives of sufferers. According to the definition of the International Association for the Study of Pain (IASP), pain is defined as an unpleasant sensory and emotional experience associated with actual or potential tissue damage [11].

Based on research, most patients with pressure ulcers or venous leg ulcers experience severe and continuous pain, especially when changing the dressing [12, 13]; therefore, the first treatment priority for many patients will be pain reduction [14]. Research has shown that uncoordinated wound care management practices and the use of outdated methods lead to high costs for health services [15]. Also, reducing the amount of pain caused by the treatment of patients’ wounds is largely affected by the treatment method and the type of dressing used. Pain from wound dressings can be controlled with careful assessment, selection of appropriate dressings, and skillful wound management [2, 11].

One of the best dressing methods is to use the Aloe Vera gel. Aloe Vera is a medicinal plant with a long history in many countries, including China, Mexico, and Greece; traditionally used for centuries as a local medicine for various diseases and skin lesions [16, 17]. Among the more than 250 species of Aloe throughout the world, the most popular and widely used species is Aloe barbadensis Miller (also called Aloe vera Linne) [18, 19]. Aloe Vera (L.) Burm. f. (Liliaceae family) is the most widely studied among four other species of Aloe, and its clinical properties and therapeutic effects have been confirmed in many diseases, especially skin disorders [20]. Aloe Vera contains polysaccharide compounds Mannose-6-phosphate and Acemannan, accelerating wound healing [21, 22]. Numerous studies and clinical trials provide significant scientific evidence showing that this plant’s effectiveness is due to the synergistic effect of several bioactive components present in it [12, 14, 15, 23]. The findings of various studies have shown that Aloe Vera has a strong analgesic and anti-inflammatory effect, and its use is effective for treating inflammation and controlling pain [24, 25]. By inhibiting the growth of various pathogenic organisms, including Escherichia coli, Aspergillus niger, Candida, Staphylococcus aureus, Salmonella, Streptococcus, and Aloe Vera gel increases tissue shelf-life [26]. Despite several case reports of Aloe Vera toxicity in humans, no controlled toxicological studies have been published [18].

Normal saline (0.9% NaCl) is currently popular as a wound-cleansing solution. This isotonic composition does not interfere with the natural healing process, does not cause tissue damage or sensitivity, and does not alter the natural bacterial flora of the skin [27]. In addition to being expensive, many synthetic drugs and dressings used to treat pressure ulcers also cause problems such as allergies and drug resistance. This situation has led researchers to search for alternative medicines, especially herbal therapies [28]. This study aimed to determine the effect of Aloe Vera gel versus saline on pain relief of pressure ulcers.

## Method

### Study design

This study is a double-blind, randomized clinical trial conducted in Valiasr Hospital in Arak, Markazi Province, in the center of Iran from May 2020 to April 2022.

Using the available sampling method, 95 patients with pressure ulcers were assessed for eligibility based on the inclusion and exclusion criteria of the study. For random allocation, people were selected by random sampling (coin toss) and placed in two groups of ten people. Then, the background characteristics of the patients in each group were evaluated. Subsequent patients who entered the study were admitted to a group that minimized the overall difference. As a result, 64 patients were selected and randomly placed in two groups: A (32 patients in the experimental group: dressing with topical use of Aloe Vera gel) and B (32 patients in the control group: normal saline dressing). In this randomized and controlled clinical trial, the researchers placed the participants in the intervention and control groups. After the completion of the intervention, data was collected by the researchers. In all stages of the intervention and data collection, patients and nurses were unaware of the people belonging to the experimental and control groups.

The inclusion criteria included people with Cate-gory II pressure ulcers with a diameter of less than 10 square centimeters and an age range of 15 to 90 years. Exclusion criteria included patients with a history of diabetes mellitus, AIDS, HIV, and other immunodeficiency such as tuberculosis, pregnant women, and failure to sign a written consent form. This research was designed and implemented according to the CONSORT guidelines (Diagram 1).

**Diagram No. 1:**
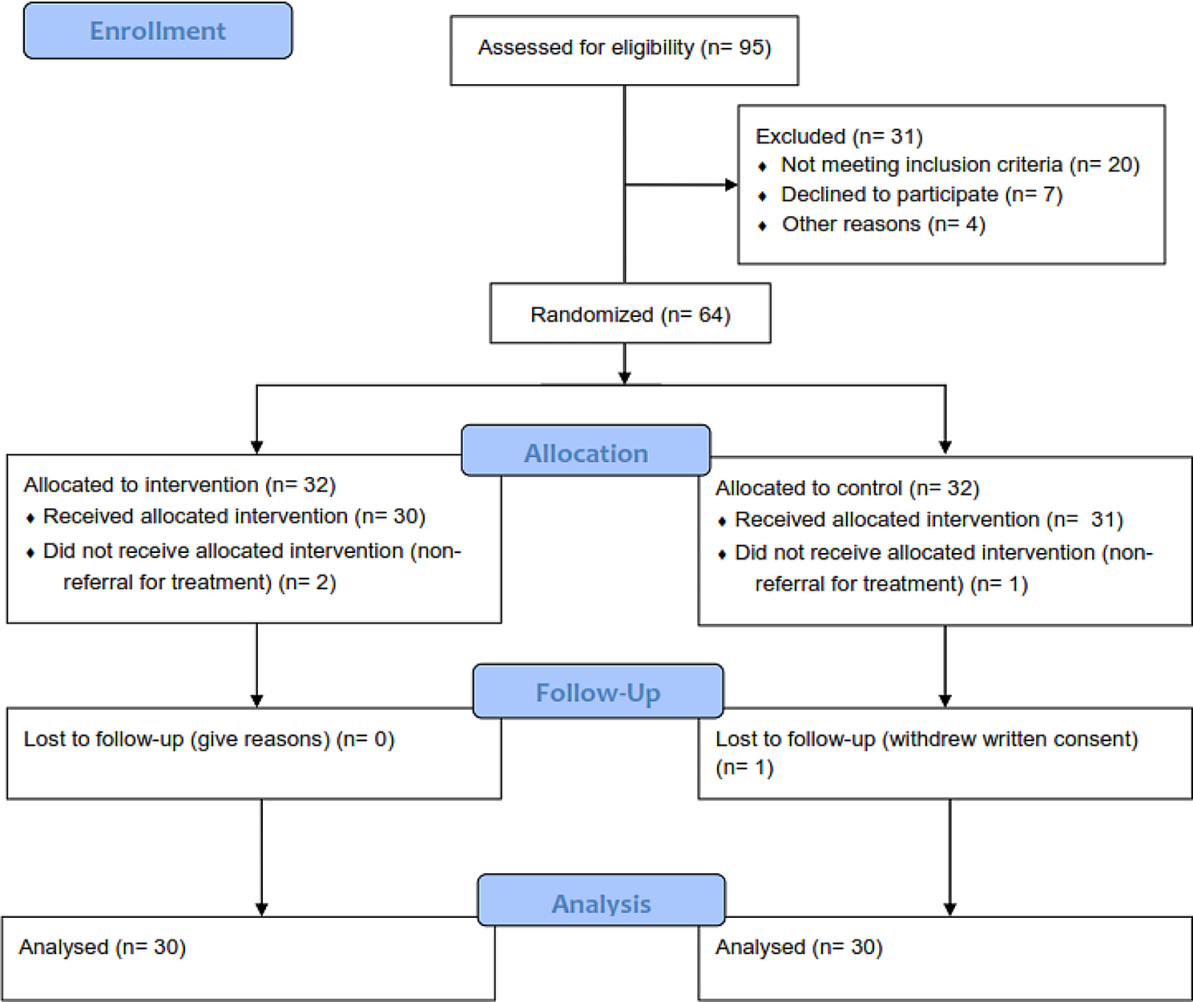
Consort -flow- diagram

### Protocol

The study protocol can be viewed at https://www.irct.ir/trial/40330.

A consistent protocol was followed for the management of patients: Keeping serum albumin > 3 g/dl and hemoglobin more than 11 g/ gm%; Deep tissue culture for systemic antibiotics of aerobic and anaerobic organisms according to culture sensitivity and according to its report (antibiotics were prescribed to patients with infected wounds); Smoking and alcohol were prohibited for patients [29].

### Plan of study

The patients were divided into two treatment groups for prospective comparative study:

- Group A (Experimental Group): Dressing with topical application of Aloe Vera gel.
- Group B (Control Group): Normal saline dressing.

The ulcers were first cleaned with normal saline for Group A patients (patients receiving Aloe Vera gel therapy) to remove the slough. Then, the already prepared Aloe Vera gel was evenly applied over the ulcers. It was then covered with a sterile cotton gauze, and the wound was tightly bandaged with a cotton roll to keep the “daily dressing” in place.

For Group B patients (control group), the ulcers were first cleaned with normal saline to remove off the slough and then covered with a sterile cotton gage, and the wound was tightly bandaged with a cotton roll to keep the “daily dressing” in place.

The Visual Pain Scale was completed before the intervention, at the end of the first week, at the end of the second week, and at the end of the third week for each patient. Then, according to the points obtained from it, the healing process of the wound was analyzed.

### Method of measurement of pain

The instrument used was the Visual Pain Scale, which was used to evaluate the subjective level of pain intensity at or near the wound site by Melzak and Katz (1994). On this standard linear scale, 0 represents no pain, and 10 illustrates the most severe pain. Scores 1, 2, and 3 indicate mild pain, 4, 5, and 6 indicate moderate pain, and 7, 8, and 9 indicate severe pain [30].

### Data analysis

Data analysis was done using SPSS 17. Descriptive statistics were used to determine the mean and standard deviation. ANOVA and greenhouse tests were used to compare the groups. The significance level was 0.05.

## Result

During the study, 64 patients with Cate-gory II pressure ulcers were included; two from the experimental group and one from the control group were excluded because of non-referral for treatment. Also, one person from the control group was excluded from the study because of withdrew written consent. The average age of the experimental group was 70.48 ± 11.33, and the control group was 69.58 ± 15.65 years.

The mean pain score of patients with pressure ulcers before and during intervention was compared in two groups. Table (1) shows that in the first evaluation (before the intervention) in the experimental group, the mean and standard deviation of the pain score was 5.3636 ± 1.13334. At the same time, it reached 1.000 in the last evaluation of this group. In the control group, in the first assessment (before the intervention), the mean and standard deviation of the pain score was 6.6563 ± 1.89412. In the last evaluation, this score reached 4.9688.

**Table 1:** Mean and standard deviation of the pain score of patients in the experimental and control groups

group	The amount of pain per day 1 (M ± SD)	The amount of pain per day 7 (M ± SD)	The amount of pain per day 15 (M ± SD)
experimental	5.3636 ± 2.13334	3.2424 ± 1.87133	1.000 ± 0.000
Control	6.6563 ± 1.89412	6.3438.6 ± 1.87133	4.9688 ± 0.87487

In the present study, the ANOVA test was used in repeated measurements, and its output showed that the average pain score in both groups had a downward trend; that is, both dressings effectively reduced pressure ulcer pain (*P* < 0.001). The greenhouse test results shown in Table No. 2 showed that the difference between the two groups was significant (*P* < 0.001).

**Table 2:** Test of intragroup effects

Variable	Mean	F	P*
The amount of pain	172.095	131.042	*P* < 0.001
Pain level	34.606	1.750	*P* < 0.001

*Greenhouse

## Discussion

The results of this study indicated the positive impact of the Aloe Vera gel on reducing the pain of pressure ulcers. Daily evaluation of the wound healing process for the experimental group showed that the wound blisters treated with Aloe Vera caused no damage to the blister membrane and blister wall while absorbing the secretions. Over time, after the appearance of pink epithelial tissue in the wound bed, the blister wound membrane was gently removed, which caused less pain. However, in the normal saline-treated group, removal of the blister wound membrane resulted in blister rupture and redness and inflammation of the repair tissue beneath the blister. It was a painful experience for the patient. It is worth noting that the use of Aloe Vera on wounds with punctured blisters caused severe pain and burning, which led to the removal of one participant from the study due to this issue. In justification of these findings, it can be said that carboxypeptidase in Aloe Vera inactivates bradykinin, a powerful factor in acute inflammatory pain. The conversion of histidine to histamine in mast cells is controlled by magnesium lactate present in Aloe Vera gel, and it is introduced as an anti-itch and pain reliever. Other studies have also shown that the blister membrane is a suitable dressing for pressure ulcers. If it can be preserved on the wound without piercing or infecting, it can prevent more profound injuries, which leads to less pain and a faster healing process [31].

The present study’s findings are broadly consistent with other Aloe Vera gel studies. Varaei et al.‘s study [32] aimed to compare the effect of Aloe Vera gel and nitrofurazone on pain caused by superficial pressure ulcer dressings, which showed that the intensity of dressing pain was significantly reduced during 72 h in both types of treatment. Still, the effectiveness of Aloe Vera gel in reducing pain was greater than nitrofurazone’s. Hekmatpou et al.‘s study on pressure ulcers in patients hospitalized in the orthopedic department showed the effect of Aloe Vera gel in preventing the increase in temperature, swelling, and skin pain in the studied areas [33]. The study of Alam Al-Hadi et al. on the effect of Aloe Vera gel on breast fissures in lactating women showed that Aloe Vera gel is effective in wound healing [34]. The study of Sabzaligol et al. aimed to evaluate the effect of Aloe Vera gel on perineal pain and showed that Aloe Vera gel reduces perineal pain after episiotomy [35]. Eshghi et al.‘s study of the effects of Aloe Vera cream on post-hemorrhoidectomy pain showed that patients had significantly less post-operative pain [36]. These results confirm the findings of the present study.

It seems that it is possible to rely on herbal treatments, including Aloe Vera, which have positive effects in many cases, reducing the costs imposed on the treatment system and the patients and causing fewer side effects for the patients under treatment.

The strength of the present study was the proper cooperation of the hospital staff to perform the intervention and follow-up of the patients. One of the study’s limitations was prolonging the study period due to the scattering of hospitalized cases with the desired disease. Also, the different experience of pain in other people can affect their self-report results.

## Conclusion

The study showed the effect of Aloe Vera gel in reducing pressure ulcer pain. According to these results, dressing with Aloe Vera gel is preferable to reducing pain during dressing changes in patients with pressure ulcers. Considering the rare side effects of Aloe Vera and society’s preference for herbal medicines, the use of this medicinal plant to reduce the pain of wounds is recommended as a complementary treatment along with other methods. Also, it is suggested that clinical trials be conducted on a wide scale and with the control of related variables to commercialize this method.

## Data Availability

The datasets generated and analyzed during the current study are not publicly available because they contain raw data from study participants, and sharing these data requires participants’ permission. But are available from the corresponding author on reasonable request.
